# Genome Editing by Grafting

**DOI:** 10.3390/ijms26199294

**Published:** 2025-09-23

**Authors:** Samuel Simoni, Marco Fambrini, Claudio Pugliesi, Ugo Rogo

**Affiliations:** Department of Agriculture, Food and Environment, University of Pisa, Via del Borghetto, 80, I-56124 Pisa, Italy; samuel.simoni@unipi.it (S.S.); marco.fambrini@unipi.it (M.F.)

**Keywords:** CRISPR/Cas, genome editing, grafting, mobile RNA, tRNA-like sequences, transgene-free plants

## Abstract

Grafting is the process of joining parts of two plants, allowing the exchange of molecules such as small RNAs (including microRNAs and small interfering RNAs), messenger RNAs, and proteins between the rootstock and the scion. Genome editing by grafting exploits RNAs, such as tRNA-like sequences (TLS motifs), to deliver the components (RNA) of the clustered regularly interspaced short palindromic repeats (CRISPR)-associated protein 9 (Cas9) system from transgenic rootstock to wild-type scion. The complex Cas9 protein and sgRNA-TLS produced in the scion perform the desired modification without the integration of foreign DNA in the plant genome, resulting in heritable transgene-free genome editing. In this review, we examine the current state of the art of this innovation and how it helps address regulatory problems, improves crop recovery and selection, exceeds the usage of viral vectors, and may reduce potential off-target effects. We also discuss the promise of genome editing by grafting for plants recalcitrant to in vitro culture and for agamic-propagated species that must maintain heterozygosity for plant productivity, fruit quality, and adaptation. Furthermore, we explore the limitations of this technique, including variable efficiency, graft incompatibility among genotypes, and challenges in large-scale application, while highlighting its considerable potential for further improvement and future broader applications for crop breeding.

## 1. Introduction

Clustered regularly interspaced short palindromic repeats/CRISPR-associated protein (CRISPR/Cas) systems are precise and efficient genome editing (GE) techniques that modify specific regions of DNA within cells [1,2]. In particular, the CRISPR/Cas9 system has become the mainstream GE technology that enables the targeted alteration of specific genomic sequences, producing double-stranded DNA breaks (DSBs) [3]. Depending on the endogenous repair pathways of DSBs, knockout and knockin are putatively possible [4]. A gene knockout is usually caused by an error in template-free end-joining pathways, such as Non-Homologous End Joining (NHEJ), which results in a small deletion or insertion. Gene knockin requires either double-stranded DNA (dsDNA) or single-stranded DNA (ssDNA) donor templates, depending on the Homologous-Directed Repair (HDR) pathways, such as homologous recombination (HR), single-strand template repair (SSTR), and microhomology-mediated end-joining (MMEJ) [5]. While Cas9 is a widely used CRISPR nuclease, other Cas proteins like Cas12a (formerly known as Cpf1) are also utilized for GE. Cas12a offers unique advantages, such as targeting different PAM sequences, generating staggered DNA breaks, and being smaller compared to Cas9. Additionally, alternative Cas proteins, such as RNA-targeting Cas13, are being actively investigated for a broad range of applications [2,6,7]. A nickase Cas9 (Cas9n) has one active nuclease domain (either RuvC or HNH) and one inactivated domain, causing it to make a single-strand break (nick) in the DNA. Using two Cas9n molecules targeting opposite DNA strands can create a double-strand break with increased specificity, reducing off-target effects [8]. Cas9n can also be linked with a deaminase enzyme to create a base editor method [9] and applied in prime editing tool, a technique that allows the introduction of specific DNA changes without creating double-strand breaks [10]. In its application, GE is revolutionizing plant breeding by providing a more efficient, precise, and faster way to develop new crop varieties with improved traits, like disease resistance, yield, and nutritional value [11,12,13,14].

GE allows the study of gene function by targeted gene knockouts or modifications to elucidate the roles of genes in various biological pathways in plants [15] and animals [16]. A nuclease inactive Cas9 [deactivated Cas9 (dCas9)] has been fused with various effector domains and applied for activation/repression of genes by creating useful methods such as dCas9-TV, CRISPR-Act3.0, and CRISPRi [17,18,19,20]. It can be used in the epigenome editing targeting specific genomic locations to modify epigenetic marks and influence gene expression [21,22]. dCas9 can also be used in genome imaging to visualize specific DNA sequences or protein interactions at targeted genomic regions [23]. Translation initiation is also an important step in controlling the activity of target proteins, involving multiple factors and regulatory elements [24]. In fact, non-coding regions of mRNAs, such as the 5′-untranslated regions (5′UTRs), contain regulatory elements in the control of protein synthesis [25]. For example, upstream open reading frames (uORFs) are important regulatory elements. Located within the 5′ UTRs, they can significantly impact the translation of the downstream main open reading frame (mORF), either by promoting or repressing it [26]. Recently, studies in *Arabidopsis* and rice have also shown that the introduction of an uORF can reduce target gene expression [27,28], suggesting a potentially promising methodology for gene silencing. In rice, Shen et al. [29] optimized the method, named *in-locus* silencing, by inserting a tailor-designed uATG-containing element (ATGE) at the primary start codon (pATG) position. In rice, the results obtained showed a substantial reduction in expression of the protein target [29].

Traditional methods for the delivery of CRISPR/Cas components into plant cells, as well as plant transformation and regeneration, are time-consuming and can raise legal issues [30]. Moreover, established methods of plant cell transformation, such as *Agrobacterium*-mediated transformation and biolistic bombardment, are not universally applicable to all crops [31]. Many plant species, as well as important cultivated genotypes within those species, have difficulty regenerating whole plants from transformed cells or tissues [32]. This regeneration is crucial for producing modified plants with heritable traits [33,34,35]. Furthermore, the generation of genomically modified plant lines using CRISPR/Cas9 without transgenes does not require crossing programs to eliminate stably integrated CRISPR/Cas9 components [36,37].

This review examines the emerging field of grafting-mediated GE. We discuss current achievements and limitations and highlight the substantial future potential of this approach for crop improvement.

## 2. The Delivery of CRISPR/Cas Components in Plant Cells

Plant cells have rigid cell walls that hinder the entry of large molecules such as DNA and proteins, including CRISPRs [38]. Therefore, there is a need to develop highly efficient, genotype-independent delivery methods that can be used in different plant species [35,38]. Several methods for delivering CRISPR reagents are being investigated, including physical (e.g., particle bombardment, electroporation, microinjection, and silicon carbide whiskers), chemical (e.g., polyethylene glycol (PEG)-mediated delivery, lipofection, and nanoparticle-mediated delivery), and biological (e.g., *Agrobacterium*-mediated delivery, protoplast-based systems, and virus-mediated delivery) approaches [39,40,41,42]. An original system involves the in planta de novo induction of meristems [43]. This approach can be particularly useful in planta transformation, where gene editing is performed directly in the plant rather than in tissue culture. It involves introducing specific developmental regulator genes, such as *WUSCHEL* (*WUS*) and *SHOOT MERISTEMLESS* (*STM*), along with gene-editing reagents, into plant tissues (e.g., using *Agrobacterium*). The introduction of these developmental regulators triggers the formation of new meristems in the treated tissue. These newly formed meristems then develop into shoots that carry the desired genetic modifications introduced by the gene-editing reagents [43]. Several studies have shown that co-delivering CRISPR/Cas9 with *WUS* and *STM* can lead to the efficient formation of new shoots and successful gene editing in various plant species [44,45,46]. It also offers a way to create transgene-free, genome-edited plants, which may be more publicly acceptable.

An approach named editing during haploid induction, often referred to as Haploid-Inducer-Mediated Genome Editing (IMGE) or HI-Edit, utilizes haploid inducers carrying CRISPR/Cas9 systems to introduce specific mutations into the genome during haploid formation [47]. IMGE can be applied to a wide range of crops, including those that are difficult to regenerate from tissue culture [48]. During embryonic development, one set of chromosomes is eliminated, and the editing machinery operates on the remaining set, resulting in haploid progeny with the targeted mutations [49]. The edited haploids are free of the CRISPR/Cas9 components, as the system is lost during haploid formation, eliminating the need for transgene removal. This accelerates the breeding process by bypassing the need for multiple generations of self-pollination to achieve homozygosity [50].

Among the methods for delivering CRISPR/Cas components, the use of ribonucleoprotein complexes and viral vectors currently appears to be one of the most promising. The delivery of CRISPR/Cas in the Cas protein + sgRNA combination (ribonucleoprotein complex, RNP) has been shown to be extremely effective, as it increases on-target activity while reducing non-specific activity [51,52]. In particular, the transient nature of RNPs, which are quickly degraded after use, minimizes the time Cas9 is active in the cell, thus reducing off-target editing [53]. RNPs can be delivered into cells using various methods, including electroporation, microinjection, liposomal, and sonication-assisted whisker delivery. Compared to plasmid or viral delivery, RNPs generally cause less cellular toxicity [54]. Notably, by directly delivering the active CRISPR machinery, RNPs bypass the need for transcription and translation, leading to immediate gene editing activity [55]. GE via RNP delivery has been successfully demonstrated in many plant species [56,57,58]. Nevertheless, this method has some disadvantages, such as the high cost, the need for protoplast culture techniques, and the impossibility of using selective agents [59]. In addition, RNPs, being large and charged, may struggle to cross cell membranes and reach the nucleus, especially in certain cell types [60]. Cas9 RNPs having a relatively short lifespan inside the cell may not be suitable for applications requiring prolonged editing [61]. Therefore, unlike plasmid vectors that provide persistent Cas9 expression, RNPs offer less control over the timing and location of editing [62]. In particular, RNPs can be susceptible to degradation by nucleases, limiting their effectiveness within the plant cell. Moreover, the lower CRISPR/Cas editing efficiency observed with RNP delivery compared to other methods may be attributed to species-specific factors and the use of plant tissues with tough cell walls, reducing the delivery to all target cells [63,64].

An alternative approach is the use of viral vectors, known as Virus-Induced Genome Editing (VIGE) [65,66,67]. Instead of stably transformed plants with the CRISPR components, VIGE employs viral vectors to transiently introduce the gRNA and/or Cas9 into plant cells. The viral vectors replicate within the plant cells, expressing the CRISPR components, but they do not integrate into the plant’s genome [68]. The current VIGE toolkit includes many RNA viruses that have been successfully applied to express sgRNA for targeted editing, although they are usually based on transgenic plants that constitutively express Cas9 nuclease [69]. The CRISPR/Cas system then introduces targeted changes to the DNA sequence at the specific location guided by the gRNA. Importantly, VIGE can induce changes in the germline, meaning that the edited DNA can be passed on to the next generation, leading to heritable changes in the plant’s traits [70]. VIGE can achieve high levels of gene editing, sometimes reaching 70% or more [66]. In addition, editing can occur relatively quickly, within 3–7 days after infection [71]. VIGE has been successfully applied to various plant species and is considered a promising approach for crop improvement and functional genomics studies [72,73,74,75]. Research has demonstrated the effectiveness of VIGE in potato and eggplant using a Potato Virus X (PVX) vector system [76]. Recently, viruses have been engineered to provide miniature CRISPR systems that enable transgene-free germline GE in *Nicotiana benthamiana* and *Arabidopsis thaliana* without the need for tissue culture or plant transformation [77,78]. The VIGE method has been successfully applied in monocotyledons. For example, VIGE has been applied in wheat by utilizing the Barley stripe mosaic virus (BSMV) vector to deliver sgRNAs [72], while the foxtail mosaic virus (FoMV) has been used for VIGE in sorghum [79].

When the plant expresses Cas9 and the virus delivers the gRNA, editing usually occurs. However, to avoid any foreign DNA integration, VIGE research has focused on identifying viruses able to incorporate and deliver both CRISPR/Cas components. Tomato spotted wilt virus (TSWV) used in *Nicotiana benthamiana* and *Capsicum* spp. is one of them [75,80]. Nevertheless, in these cases, edited leaf portions are still required in vitro culture to originate edited plants. Other studies have aimed to obtain transgenic-free plants by avoiding in vitro culture. In *N. benthamiana*, the co-inoculation of Tobacco ringspot virus (TRSV) delivering Cas9 and TRV delivering gRNA-*FT* enabled virus-mediated heritable gene editing [81]. More recently, in one of the world’s most important crops, barley yellow striate mosaic virus (BYSMV) was modified to deliver the CRISPR components in wheat cultivars. In this system, both RNAs were fused to tRNA-like structures, allowing the production of heritable gene editing in different wheat cultivars [82].

Nevertheless, several problems need to be addressed before VIGE can be adopted on a large scale. For instance, most plant viruses are not seed-borne, and those transmitted through seeds may not have sufficient carrying capacity. Removing viral proteins involved in the transmission to increase carrying capacity, but this comes at the cost of mobility and requires tissue culture, which is problematic for many crops [66]. If editing is uncompleted before a cell divides, it can lead to genetic mosaicism, where only a subset of cells carries the edited gene while others remain unedited [83]. Many studies have shown that while somatic cells are edited, the edits are not passed on to the next generation, indicating a difficulty of virus or sgRNA distribution in the germline [84]. In addition, some viral vectors have a narrow host range, limiting their application to a specific set of plant species and genotype [85]. Despite these limitations, VIGE holds great promise for crop improvement and has the potential to revolutionize plant breeding. Ongoing research is focused on overcoming these challenges and expanding the application of VIGE to a wider range of crops [86].

Transgene-free GE is crucial for crop improvement because it minimizes the risk of unintended genetic changes in the edited plant, unlike methods that integrate foreign DNA into the genome. Recently, Qiu et al. [87] have developed an innovative method, which provides an mRNA delivery system into plant cells by improving the translation efficiency of in vitro transcribed mRNA and its stability during particle bombardment-mediated transformation. Specifically, it was found that poly(A) tail extension, 5′UTR optimization, and mRNA coating with protamine had synergistic effects in improving the editing efficiency of knockout genes and base changes from C (cytosine) to T (thymine) and A (adenine) to G (guanine) in both rice and wheat experiments [87].

## 3. Genome Editing by Grafting (GEG)

It is known that in plants, RNA molecules can move from roots to shoots, and this long-distance transport plays a role in plant development and signaling [88]. These molecules include messenger RNAs (mRNAs), small RNAs [sRNAs, like microRNAs (miRNAs) and small-interfering RNAs (siRNAs)], transfer RNAs (tRNAs), and long non-coding RNAs (lncRNAs). This movement can occur through the phloem and also via cell-to-cell transport through plasmodesmata. In the vascular system, the phloem contains physiologically active companion cells and sieve elements, forming a complex [89], which, along with plasmodesmata, establishes a transport network that supports the long-distance movement of RNA between different plant organs [90]. Notably, the discovery of mobile mRNA challenges the traditional view of mRNA as solely a local signal, highlighting its role in systemic communication and coordination within a plant. It has also been shown that some immobile mRNAs are activated to move long distances under conditions of nutritional deficiency [91,92], and their mobility in the shoot is also positively affected by their expression levels [93,94].

Grafting experiments in plants have significantly advanced the study of mobile RNA molecules, particularly in understanding how these molecules move between different plant parts and how they influence plant development and responses to environmental stress [95]. Plant grafting is an ancient, vegetative, asexual plant propagation technique used to join parts of two or more plants so that they grow as a single plant. It involves attaching a scion (a shoot or bud) to a rootstock (the base of a plant with roots). This method is used to combine desirable characteristics of different plants, such as disease resistance, adaptability to specific soil conditions, or fruit and seed quality [96,97].

Remote signals represented by mRNAs and sRNAs transmissible to the graft are now emerging as new mechanisms that regulate the nutritional and developmental relationships between root and shoot and may play a key role in graft physiology [97,98,99]. Various research on grafted plants has shown that thousands of mRNAs move over long distances between plant tissues [100,101,102,103], potentially acting as signals [104]. These RNA signals play a role in several physiological and developmental processes, such as coordinating growth and development across different organs, leaf development [105], meristem maintenance and root development [106], flowering time, tuber formation, ripening of fleshy fruits, and responses to environmental cues, like drought, temperature changes, and pathogens [107,108,109]. For example, potato *StBEL5* mRNA, from the gene encoding for BEL1-like transcription factor (TF), is transported from leaves to roots and stolons through the phloem to regulate tuberization [110,111].

Notably, a signal of water deficiency was first sensed by the root tissue of the rootstock (*Solanum pennellii*) and then transmitted to the leaf tissues of the scion (*Solanum lycopersicum*) in an interspecific grafting system subjected to drought stress. This drought signal was transmitted to the leaf tissue, which reduced the photosynthetic and transpiration rates, thereby reducing plant water loss and enhancing the drought tolerance of the grafted plant [112]. The authors detected 61 upwardly and 990 downwardly mobile mRNAs (mob-mRNA) in the grafted tomato system. The results indicated that mob-mRNAs were involved in RNA binding, photosynthesis, response to heat, translation, and carbon metabolism pathways [112].

Several studies have also suggested that some specific motifs or structural sequences may affect the mobility of mRNAs in grafted systems. These motifs may affect how mRNA interacts with RNA-binding proteins (RBPs) or how it is packaged for transport [113]. For example, it was found that a tRNA-like structure in the 3′ UTR of some mRNAs is sufficient for long-distance movement [92]. Specific sequence motifs in mRNAs can be recognized by certain RBPs, and these interactions can affect the mRNA’s mobility. For instance, a poly-pyrimidine sequence in the 3′ UTR of the *StBEL5* mRNA interacts with polypyrimidine tract-binding proteins (PTBs) [111]. It has also been observed that truncated sequences of mobile endogenous mRNAs, such as the *FLOWERING LOCUS T* (*FT*) and *GA-INSENSITIVE* (*GAI*) of *Arabidopsis thaliana*, are unable to permeate through graft complexes [114,115,116]. Besides *FT* and *GAI*, other mobile mRNAs like *CmNACP*, encoding a NAC domain protein in melon, and *LeT6*, encoding a tomato *KNOX* gene, also have specific sequence motifs that facilitate their movement [105,117]. Interestingly, cytosine methylation was found to be a key factor in the mobility of mRNAs in plants. Among the mob-mRNAs detected in *Arabidopsis* grafting assays, cytosine methylation was significantly enriched. The movement of *TRANSLATIONALLY CONTROLLED TUMOR PROTEIN 1* (*TCTP1*) and *HEAT SHOCK COGNATE PROTEIN 70.1* (*HSC70.1*) transcripts from shoot to root was abolished in the absence of cytosine methylation [118].

**Figure 1:**
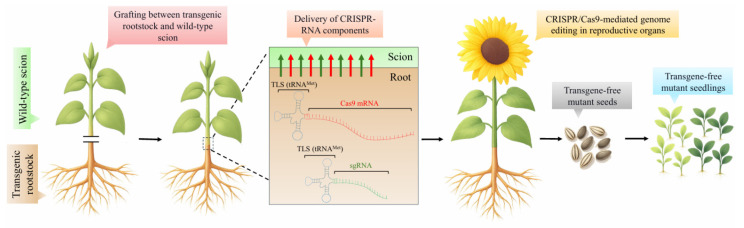
Using mobile CRISPR-RNA, inter- or intra-specific grafting between sunflower scion and transgenic rootstock. Mobile Cas9 and sgRNA RNAs fused to tRNA-like sequence (TLS) motifs moved from the root to the wild-type shoot. The CRISPR complex induced targeted genome editing (GE) in the distal parts and reproductive organs of the grafted scion. Red arrows correspond to the TLS (tRNA^Met^)-Cas9 mRNA, while green arrows indicate TLS (tRNA^Met^)-sgRNA. Transgene-free mutant seeds (light brown) and consequently mutant seedlings (light green) are obtained.

Based on previous discoveries, a novel approach of GE uses grafting to deliver Cas9 mRNA and sgRNA, the principal actors of the CRISPR/Cas system, from transgenic rootstocks to wild-type scions, generating transgene-free mutant seedlings [119]. Although this technique may seem laborious, especially for those species and cultivars where classical approaches and VIGE can work well, it has excellent advantages for plants that are recalcitrant to in vitro culture or tree species that need to maintain their heterozygosity. This novel approach is summarized in Figure 1, which illustrates the possible application of the technique in sunflower (*Helianthus annuus* L.), a species notoriously difficult to regenerate and for which gene-editing approaches remain particularly challenging [120].

Yang et al. [119] are the pioneers using the grafting concept to perform edited plant lines with heritable modifications. They demonstrated that both intra- and inter-species grafting between wild-type *Arabidopsis thaliana* and *Brassica rapa* could produce heritable edits without direct transformation of the scion.

In their experiment a wild-type scion is grafted onto a transgenic rootstock. The rootstock expresses CRISPR-RNA components (Cas9 mRNA and sgRNA) fused with tRNA-like sequences (TLS). The TLS is essential for the transport of the CRISPR/Cas9 components from rootstock to scions [119]. Once fused with TLS, the CRISPR/Cas9 components are transported from the rootstock to the scion, potentially distributed throughout the plant.

TLS was first identified more than 50 years ago in the genome of turnip yellow mosaic virus (TYMV) [121]. The function of TLS remained unclear for a long time, also because its role varies between different virus types. However, it appears to be able to direct host ribosomes to translocate internally to the translation initiation site, enhancing translation and controlling ribosome/replicase trafficking in the TYMV system. Distinct TLSs contribute to the movement of mRNAs from transgenic roots to leaves and from leaves to flowers or wild-type roots. In particular, proteomic data on grafted plants indicate the presence of proteins from mobile RNAs, indicating the possibility that they are translated at the target site. Furthermore, Zhang et al. [92] demonstrated that dicistronic mRNA:tRNA transcripts were frequently produced in *A. thaliana* and are enriched in the graft-mobile mRNA population. These results suggested that tRNA-derived sequences with predicted stem-bulge-stem-loop structures were sufficient to mediate mRNA transport and appeared to be indispensable for the mobility of a large number of endogenous transcripts that could move across graft junctions [92]. The authors showed that adding TLS to the *DISRUPTION OF MEIOTIC CONTROL 1* (*DMC1*) gene was sufficient to mobilize *DMC1* from stocks grafted into the shoot apex, where it induced male sterility.

Using intra-species grafting in wild-type *Arabidopsis thaliana*, Yang et al. [119] built two gRNAs to cause a genomic deletion of approximately 1000 base pairs in the *NITRATE REDUCTASE1* (*NIA1*) gene, which encodes the cytosolic minor isoform of nitrate reductase (NR), resulting in a *nia1* knockout. *NIA1* is involved in the first step of nitrate assimilation, contributing to about 15% of the nitrate reductase activity in shoots [122]. On NH_4_-deficient soils, *nia1* mutants developed an easily visible chlorotic phenotype over time, characterized by smaller plants than the wild type [123]. Two types of TLS were used for tagging: TLS1, an intact methionine-tRNA (tRNA^Met^) sequence, and TLS2, an altered and shorter methionine-tRNA (tRNA^Met-ΔDT^) sequence. However, no significant difference was observed in the mobility of Cas9 transcripts and/or sgRNAs tagged with TLS1 or TLS2 [119].

The chlorotic phenotype was observed in some differentiated leaves on grafted scions of plants grown on NH_4_-deficient soil as early as 14 days after grafting, suggesting that both Cas9-TLS and sgRNA-TLS transcripts were successfully transported into the scion. In addition, RT-PCR analysis and genotyping showed that the transported transcripts and genome modifications were present in both flowers and siliques of grafted plants, demonstrating that the fusion transcripts were active in the reproductive tissues. The offspring analysis of the wild-type grafted *Arabidopsis* plants revealed homozygous *nia1* mutants with frequencies of 1.17–1.41 per 1000 seeds, indicating that this transgene delivery method generated heritable changes in *Arabidopsis* [119]. Therefore, the CRISPR/Cas9 system guided by the gRNA can then target and edit specific DNA sequences in the scion. As the CRISPR/Cas9 components are not integrated into the scion’s genome, the resulting seeds carry only the edited DNA, without any trace of the foreign genes, resulting in a significant advantage for regulatory and consumer acceptance. The results demonstrated that the TLS motifs enhanced the mobility of CRISPR/Cas9 components, allowing for efficient editing in various tissues, including reproductive tissues for heritable changes.

In addition, Yang et al. [119] created two gRNAs, g*Venus1* and g*Venus2*, with and without fused TLSs, to confirm whether this mobile genomic editing system also worked on other gene targets. *Venus* is an improved version of the yellow fluorescent proteins (YFP), a variant of the green fluorescent protein (GFP), from the jellyfish *Aequorea victoria* [124]. In the experiment, transgenic scions containing the *35S_promoter_*::*H2B-Venus*::*35S_terminator_*::*BastaR* cassette were grafted on rootstock expressing Cas9-TLS and g*Venus*-TLS. The loss of *Venus* fluorescence was demonstrated in 7 of 1557 seedlings in the progeny, showing the presence of homozygous gene changes in about 0.45 percent of the plants. These results indicated that genomic editing by grafting was functional even with alternative gRNA sequences [119]. In a subsequent experiment, Yang et al. [119] assessed whether the mobile Cas9-TLS and sgRNA-TLS constructs were also transported in an important crop species, *Brassica rapa*, grafted onto transgenic *Arabidopsis* roots. The mobile and non-mobile sgRNAs were again designed to target the *NIA1* gene. The results showed that the TLS motif effectively promoted the long-distance mobility of Cas9 transcripts and gRNA from *Arabidopsis* rootstocks to *B. rapa* shoots. Furthermore, PCR analysis and sequencing demonstrated genomic changes in four of six siliques and four of six flowers of *B. rapa* plants grafted onto *Arabidopsis*. The results suggest that both the Cas9-TLS and g*NIA1*-TLS constructs are transported in sufficient quantities and are functional for GE in a heterografted crop plant [119].

All the GE techniques have advantages and disadvantages, and considering the large number of species and genotypes, the development of new approaches such as GEG further expands the toolbox for potential applications (Table 1). Using these methods individually or in combination can broaden the range of plant species amenable to GE, enabling faster and more efficient applications.

The combination of grafting and mobile CRISPR/Cas9, facilitated by TLS motifs, offers a powerful and versatile tool for transgene-free genome-edited plants, with broad implications in numerous species for both basic research and practical applications in agriculture [125,126]. It can be used to develop new varieties with improved characteristics, such as disease resistance, stress tolerance, and higher nutritional value [127]. Moreover, it can facilitate research on gene function and plant development by allowing targeted modification of specific genes.

## 4. Existing Gaps in the Implementation of the GEG

The applicability of the GEG technique is closely linked to the success of intra- and inter-species grafting between genotypes prone to modification (rootstocks) and genotypes recalcitrant to genetic transformation (scions). Intra-grafting is generally easier than interspecies grafting due to greater taxonomic and genetic compatibility. The main difficulties in inter-species grafting stem from physiological and biochemical incompatibilities that disrupt vascular connection and nutrient transport between the rootstock and scion, leading to poor growth and plant death [98,128,129]. In addition, metabolic interference can result in starch accumulation above the graft union and root starvation, similar to girdling [98,128]. Grafting can also cause imbalances in hormonal signals, particularly auxin, which are crucial for wound healing and the proper development of vascular tissues [98,130]. Finally, long-distance protein, mRNA, and small RNA (like miRNAs and siRNAs) signals between the scion and rootstock can be disrupted, affecting nutrient and developmental communication [109]. Therefore, improving inter- and intra-species grafting techniques is essential for the success of GEG in both herbaceous and woody species. Recent interesting reviews have validly discussed the advances and challenges still to be explored in grafting in different plants [97,131,132,133].

The improvement of grafting techniques in many monocotyledon species is an illustrative example. GEG could be a powerful tool for improving monocotyledons that comprise the world’s most important crop species. By enabling precise modifications to plant DNA, it offers the potential to enhance yield, disease resistance, nutritional value, and stress tolerance, contributing to food security and sustainable agriculture [6]. Moreover, biotechnological improvement of monocots is often hampered by the lack of efficient regeneration systems, requisite wound responses, and low cell totipotency. While some plant combinations are not compatible for grafting, especially within the monocot species, research is ongoing to expand the range of successful grafts. Reeves et al. [134] showed that intra- and inter-specific embryonic hypocotyl grafting was possible in all three groups of monocotyledons: commelinids, lilioids, and alismatids. They were able to successfully graft pearl millet scions onto wheat rootstock, wheat scions onto sorghum rootstock, rye scions onto sorghum rootstock, rice scions onto pearl millet rootstock, rice scions onto sorghum rootstock, and wheat scions onto oat rootstock. This discovery in monocot species identified the mesocotyl as the meristematic tissue responsible for enabling grafting. Overall, the results of Reeves et al. [134] offer the possibility of the GE of plant species with low regeneration that are difficult to propagate using mobile CRISPR-RNA.

Advances in genetic analysis and the application of plant hormones (auxins and cytokinins) help in selecting compatible rootstock–scion combinations and promoting faster healing at the graft site in several intra- and inter-species grafts. Understanding these hormones has led to improvements in grafting techniques [97]. Additionally, biotechnology is being employed to enhance grafting success rates. Genetic editing tools, such as CRISPR, offer the possibility of modifying the genes of the rootstock and scion, improving compatibility, disease resistance, and grafting success [135].

In the experiments conducted by Yang et al. [119], CRISPR-RNA components (Cas9 mRNA and sgRNA) were fused with tRNA-like sequences (TLS1 or TLS2). Currently, little is known about the factors that influence mRNA mobility [136]. Understanding the mobility capacity of mRNAs offers the possibility of investigating potential applications for the use of transcript carriers in GEG techniques as alternatives to TLSs. For example, one potential route for mRNA movement could take the form of extracellular vesicles (EVs), which are small lipoprotein membrane-derived structures that can contain or display on their surface proteins, nucleic acids, and lipids [107,137,138]. However, although the prospect of EVs driving RNA and protein movement is appealing, in only a few woody plants was the presence of EVs in the vascular system documented [139]. Therefore, further studies are needed to determine whether EVs play a key role in mRNA movement with a view to their use as carriers of CRISPR-RNA components.

Movement proteins (MPs) are produced by RNA viruses, forming RNP complexes that promote intercellular transport and thus the systemic spread of viruses [136]. Plants have proteins that may function similarly to MPs. These RNA-binding proteins carry plasmodesmata localization signals that are sufficient to mark a transcript for intercellular trafficking [140]. Examples include the RNA PHLOEM PROTEIN 16 (PP16) complex in *Cucurbita max* and the TF KNOTTED1 protein-mRNA complex [106,141,142]. Although MPs and viroids present an intriguing mechanism for targeting plasmodesmata, which in turn facilitates long-distance transport of RNAs, the expansion of this targeting for long-distance mobile mRNA in plants is limited to a few examples [136]. *GAI1* provides an interesting perspective on mRNA movement. This mRNA is capable of moving from grafted rootstocks to the shoot apex, influencing the shape of emerging leaves [143]. Interestingly, the RNA sequence of *GAI1* was both necessary and sufficient to facilitate the movement of *GFP* mRNA [114]. Specifically, both the *GAI1* coding sequence and the 3′ UTR region are sufficient for mobility [114,143]. Not all mRNAs studied functionally are consistent with these findings, highlighting the complexity of long-distance mRNA transport. For example, it has been hypothesized that the 50-kD phloem RNA binding protein (RBP50) facilitates long-distance RNA transport by binding to its own mRNA via a CUCU polymer-pyrimidine domain in both pumpkin and potato [111,144]. This domain is not significantly enriched in mobile mRNA datasets among other grafted species, suggesting that it may be a species-specific mechanism [118]. However, some data on the mobility of many mRNAs have not always been confirmed with certainty. Recently, Paajanen et al. [145] performed a meta-analysis of existing datasets related to mobile mRNA, considering the associated bioinformatics pipelines. Taking into account technological noise, biological variation, potential contamination, and incomplete genomic assemblies, the authors found that a significant percentage of currently annotated mobile transcripts were not statistically supported by available RNA-seq data [145]. This study suggests that some aspects of mRNA communication in plants should be treated with extreme caution.

Further research on mRNA mobility in plants is desirable, but current knowledge suggests that tRNA-like sequences are still the best carriers for transporting CRISPR/sgRNA components for GE via grafting.

## 5. Future Perspectives

In this review, we aim to highlight the power of the GEG method, which enables overcoming the bottleneck represented by the inability to undergo in vitro regeneration in many species. Given the enormous potential, we wanted to share this technique in its infancy, emphasizing certain aspects related to the improvement of grafting methods. Furthermore, studies on mRNA mobility need to be further investigated, also with a view to identifying alternative carriers to TLS for CRISPR/sgRNA transcripts.

Many clonally propagated crops benefit from heterozygosity, which can lead to increased vigor, yield, and resilience to environmental stress [146]. By carefully managing the propagation process and selecting plants that closely resemble the original heterozygous genotype, breeders can effectively maintain the desired characteristics of a clonal cultivar over time. Genetic transformation, cisgenesis, and GE are methods that, when applied to highly heterozygous agamic-propagated species (e.g., grapevine, apple tree, and peach tree), could offer the best opportunities for genetic improvement, if not for the legislative obstacles that prevent their application in many countries [147]. GEG offers one of the best application opportunities for GE in clonally propagated crops, especially for perennial crops with long juvenile phases. It is expected that the fruit phenotype can be seen in 2–3 years, and the problem of difficult genetic transformation can be solved, which can greatly improve the efficiency of research and breeding in these species.

**Figure 2:**
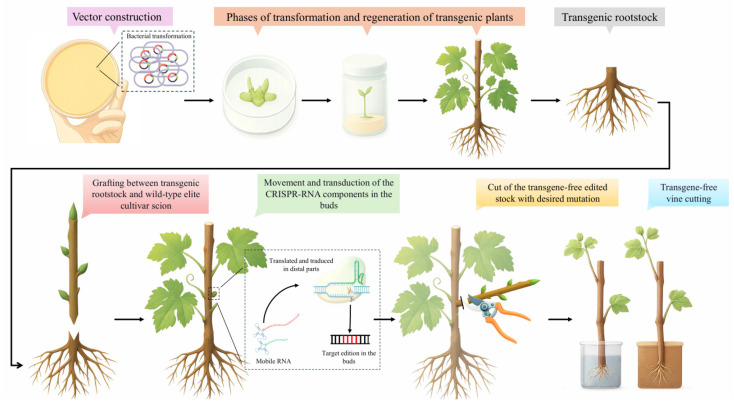
GEG in grapevine. Vectors expressing Cas9-TLS and sgRNA-TLS are cloned and transferred into an easily transformed grapevine genotype. After in vitro culture, the transgenic rootstock is grafted onto a wild-type elite cultivar scion. The CRISPR-RNA components are translated and transduced in the distal parts. The transgene-free stock is rooting, producing the identical elite cultivar scion but with the new desirable characters.

A representative plant that requires new techniques to improve several traits is the grapevine (*Vitis vinifera* L.). Almost all the studies on grapevine are applied through embryogenic calli (EC) or protoplasts isolated from EC. Unfortunately, EC is limited only to a few varieties, such as Sugraone, Crimson Seedless, Thompson Seedless, and Chardonnay [148]. This issue involves difficulties in gene transfer, tissue culture for regeneration, genetic transformation, and GE in genotypes of high agronomic interest [149]. The potential of GEG could solve this specificity to transformation by using genotypes that show high regeneration rates, grafted with elite cultivars to produce new genotypes with desirable traits (Figure 2).

In these species, even when successful transformation and editing occur, the resulting plants may be chimeric, meaning they contain a mix of edited and unedited cells, which can hinder the expression of the desired trait [150]. Nevertheless, in vegetatively propagated species, chimerism can be managed through appropriate pruning and cutting of edited plant parts. This technique allows for the isolation and propagation of stable, mutated sectors within the plant, potentially leading to the development of new cultivars.

GE also offers a powerful tool for plant conservation, including for rare and endangered species. It allows for targeted modifications to plant DNA, potentially enhancing their resilience to environmental stressors, disease resistance, and overall fitness, thus aiding in their survival and recovery. However, rare and endangered species can be difficult to manipulate in vitro due to various biological and logistical challenges. These challenges may stem from several factors as well as the unique biology of the species, the limited availability of samples, the complexity of their development, the lack of basic knowledge about their reproductive cycles, the cell behavior, and their response to different in vitro conditions. Therefore, developing and implementing in vitro protocols for regeneration and genetic transformation often requires specialized equipment, media, and expertise, which can be expensive and time-consuming [151]. Mobile CRISPR-RNA can be optimized by developing a mutant rootstock in those species that can be regenerated easily. Unmodified scions of rare and extinction-threatened species can be grafted onto mutant rootstocks containing mobile CRISPR-RNA to improve biotic and abiotic tolerance, reducing the risk of extinction [127].

Some economically important crops, such as cocoa, coffee, sunflower, cassava, avocado, and many trees and fruit crops, show major obstacles in the application of in vitro techniques (regeneration and genetic transformation), and within species there are many differences in the expression of cell totipotency among different cultivars (e.g., maize, grapevine, common wheat) [151,152,153,154,155]. In these species, GEG may prove to be an extremely promising technique.

The mobile RNA technique for GE would be useful for cultivated species that are recalcitrant to genetic transformation and characterized by narrow genetic variability, a significant problem because it limits their ability to adapt to changing environmental conditions, resist diseases, and ultimately, survive long-term. Crops with low genetic diversity have a smaller buffer when it comes to evolving to their ever-changing environment. For example, the Cavendish banana, the most commonly consumed type, is a sterile clone, making it highly susceptible to diseases like Panama disease and changing climate conditions [156]. While potatoes have some genetic variability, many cultivated varieties are derived from a small number of wild ancestors, making them vulnerable to diseases like late blight [157]. Modern wheat varieties are often highly uniform due to selective breeding for specific traits, potentially impacting their ability to adapt to environmental changes and leaf rust disease [158]. Hybrid maize varieties can have a narrow genetic base, making them susceptible to diseases and pests [156].

Although the technique appears promising, and we are only at the beginning, the results from the only available study [119] indicate that editing is heritable but with a low frequency. Thus, given its great potential, efforts must be made to improve this system. The production of the CRISPR-RNA in the rootstock is crucial; for this reason, building vectors containing specific promoters for each species is essential. Furthermore, in the last few years, several Cas proteins have been discovered. Using a smaller comparing Cas9, such as miniature Cas12 [159], OMEGA effector TnpB, and Fanzor (Fz) [160], could improve transport and translation in the distal and reproductive portions of the scion.

Furthermore, advances suggest the potential for combining GEG with inducible systems and epigenetic regulation. For example, when coupled with heat-inducible promoters, such systems allow precise environmental control over gene editing [161,162]. Moreover, the use of Cas9 fused to epigenetic effectors allows reversible gene regulation without permanently altering the DNA sequence [163]. Thus, inducible expression of mobile CRISPR/Cas9 from rootstocks, triggered only upon environmental changes or applied molecules, can enable tightly regulated editing events in scion tissues. This is particularly relevant for applications where transient activation is desired, such as inducing stress tolerance during specific growth stages or conferring temporal resistance to pathogens.

In essence, GEG can be applied to a wide range of plant species, including those that are difficult to transform using traditional methods. The combination of grafting and mobile CRISPR/Cas9, facilitated by TLS motifs, offers a powerful and versatile tool for transgene-free plant GE, with broad implications in numerous species for both basic research and practical applications in agriculture [125,126]. Moreover, it can facilitate research on gene function and plant development by allowing targeted modification of specific genes. In addition, the use of a transgenic rootstock expressing CRISPR/Cas components provides a system for studying fundamental processes and gene function and regulation in a graft-compatible manner, without the need for traditional breeding or the generation of transgenic scions. It also provides a powerful tool for dissecting the complex interactions between rootstock and scion and understanding how they influence each other’s gene expression and overall phenotype. Understanding rootstock–scion interactions, thanks to GEG, can lead to the development of improved crops with enhanced traits like yield, disease resistance, and tolerance to environmental stress.

## Figures and Tables

**Table 1:** Comparison between GEG and other GE techniques.

Method	DNA Integration	Use of Viral Vectors/Bacteria	Main Advantages	Main Disadvantages
Genome editing by grafting (GEG)	No	Yes (only in the rootstock)	- Transgenic-free mutants. - Applicable to recalcitrant and difficult plants, such as trees, to maintain their heterozygosity. - New technique with great potentialities for improvement.	- Require in vitro culture of the rootstock. - Require graft compatibility.
Virus-Induced Genome Editing (VIGE)	No	Yes	- Transgenic-free mutants. - Avoids in vitro culture (not always). - Fast.	- Limited cargo size (especially RNA viruses). - Not all viruses reach shoot apical meristems. - Host–virus specificity required.
*Agrobacterium*-mediated transformation	Yes	Yes	- Widely used and well-optimized in both monocot and - eudicot species. - Can deliver large sequences. - High efficiency in many species.	- Requires in vitro culture and selection with potential somaclonal variation. - Time- and cost-consuming. - Higher likelihood of foreign DNA integration.
Biolistic bombardment	Yes/No	No	- Can be used for species recalcitrant to *Agrobacterium*. - Directly delivers DNA, RNA, or proteins.	- Random and multiple copies integration. - Requires in vitro regeneration. - Time- and cost-consuming.
Direct physical delivery (PEG-mediated uptake, microinjection, electroporation)	No (unless DNA is delivered)	No	- Can deliver DNA, RNA, or RNPs. - Precise control over cargo. - Minimal off-target risk.	- Often requires protoplasts or specialized equipment. - Regeneration from single cells is challenging.
